# The maxillary canal in *Cynognathus* and *Diademodon* (Cynodontia, Cynognathia)

**DOI:** 10.1007/s00114-026-02133-z

**Published:** 2026-07-17

**Authors:** Julien Benoit, Valentin Buffa, Michael O. Day

**Affiliations:** 1 Evolutionary Studies Institute, University of the Witwatersrand, Private Bag 3, WITS, Johannesburg, 2050 South Africa; 2 Department of Paleontology, University of Zurich, Karl-Schmid-Strasse 4, Zurich, 8006 Switzerland; 3 Fossil Reptiles, Amphibians and Birds Section, Natural History Museum, Cromwell Road, London, SW7 5BD UK

**Keywords:** Trigeminal nerve, Phylogeny, Cynodontia, Triassic, Therapsida

## Abstract

The maxillary canal system in *Cynognathus* and *Diademodon* is described. The hypothesis that expansion of the maxillary sinus represents a synapomorphy of the clade Cynognathia is supported. A trend towards the reduction of the inferior palpebral ramus is proposed.

## Introduction

Previous assessments of the evolution of the maxillary canal (neurovascular canals for the maxillary branch of the trigeminal nerve) in cynognathians were limited by the absence of data for early-diverging taxa (Franco et al. 2021; Pusch et al. 2024; Kerber et al. 2024b; Medina et al. 2025; Roese-Miron et al. 2025). Here, we describe, based on CT data, the maxillary canals of *Cynognathus crateronotus* and *Diademodon tetragonus* from the Burgersdorp Formation (Anisian) of South Africa. This contribution highlights characters that will help improve (i) scoring of non-mammalian synapsid phylogenetic matrices that include maxillary canal characters (e.g., Benoit et al. 2022; Pusch et al. 2024), (ii) the identification of isolated maxillae (Tolchard et al. 2021), and (iii) understanding of the possible origins of whiskers among non-mammalian cynodonts (Benoit et al. 2016, 2020; Kerber et al. 2024a; Medina et al. 2025).

## Materials and methods

### *Diademodon tetragonus*

BP/1/3757, an isolated snout; scanned by Gideon Chinamatira at the Evolutionary Studies Institute, Johannesburg, South Africa, using a Nikon Metrology XTH 225/320 LC CT system. Voxel size: 0.110 mm.

## *Cynognathus crateronotus*

BP/1/3755, a complete skull; scanned by Elrentia Bussy at the Wits Donald Gordon Clinic, Johannesburg, South Africa, using a Philips Brilliance 64 slice CT scanner. Voxel size: 0.443 mm.

NHMUK PV R 3604, an isolated right maxilla; scanned by Agnese Lanzetti at the NHMUK, London, using a Nikon Metrology HMX ST 225 CT system. Voxel size: 0.051 mm.

Segmentations of the maxillary canal systems in these specimens followed the methodology introduced by Benoit et al. (2016), using Avizo 2021 (Thermo Fisher Scientific, Hillsborough, OR, USA).

Anatomical abbreviations: C, upper canines; CA, caudal alveolar; EN, external nasal; IN, internal nasal; IP, inferior palpebral; MA, median alveolar; MS, maxillary sinus; Orb, orbit; RA, rostral alveolar; SL, superior labial.

Institutional abbreviations: BP: Evolutionary Studies (formerly Bernard Price) Institute; NHMUK: Natural History Museum of the United Kingdom, London.

## Descriptions

### *Cynognathus crateronotus*

**Fig. 1:**
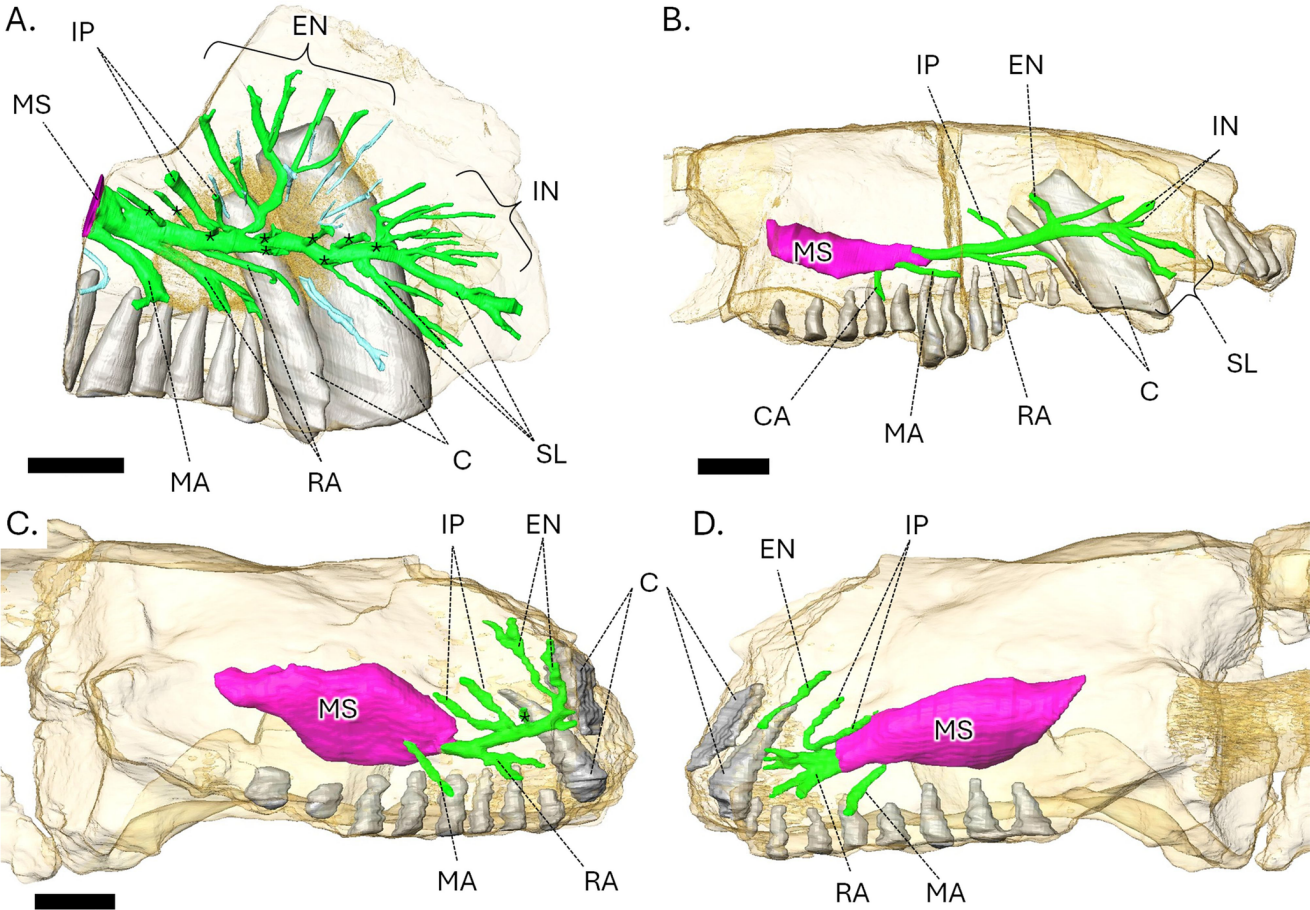
3D reconstruction of the he maxillary canal systems of *Cynognathus* and *Diademodon* in lateral view. **A**, right maxillary canal system of *Cynognathus* based on NHMUK PV R 3604; **B**, right maxillary canal system of *Diademodon* based on BP/1/3757; **C**, right and **D**, left maxillary canal system of *Cynognathus* based on BP/1/3755. *, horizontal canals (in **A** and **C**). Scale bar = 2 cm. Canals in light blue (in **A**) do not connect to the maxillary canal

Of the two specimens, NHMUK PV R 3604 is the only one that preserves branches of the maxillary canal anterior to the EN ramus (i.e., the part related to the infraorbital nerve in mammals, Benoit et al. 2020) (Fig. 1A). Anterodorsal to the level of the upper canine, the IN ramus is the most extensively branched canal. It bears ten branches organised into two clusters arranged in an anteroposterior direction: an anterior cluster comprising four branches and a more posterior one comprising six branches. Three branches located immediately ventral to the IN ramus may be identified as belonging to the SL ramus (Fig. 1A). They are oriented anteroventrally at about 30° to the horizontal. The two anteriormost branches are further subdivided into three smaller canals leading to foramina located anterior to the upper canine, whereas the posterior most branch leads to a single foramen located at the level of the middle of the canine. There are nine horizontally oriented canals branching directly from the main trunk of the maxillary canal, which have no equivalent in other cynodonts (marked * in Fig. 1A, C). Six of these canals are located anterior to the EN ramus (on the infraorbital branch), whereas the remaining three canals are located posterior to it.

The EN canal forms a complex of ramifications that branches off dorsally from the main trunk of the maxillary canal at the level of the upper canine (Fig. 1A, C). In both NHMUK PV R 3604 and BP/1/3755, it bears two main branches that further subdivide dorsally into minor branches. In NHMUK PV R 3604, where the EN canal is completely preserved and imaged at higher resolution, these ramifications are connected to six foramina on the lateral surface of the maxilla (Fig. 1A). Some additional isolated branches identified in NHMUK PV R 3604 (shown in blue in Fig. 1A) may also belong to the EN ramus but cannot be identified as their roots could not be traced. Posterior to the EN ramus and anterior to the MS, there are two additional posterodorsally oriented branches arising from the main trunk of the maxillary canal in both specimens (Fig. 1A, C, D). Comparable ramifications in a similar position to those present in *Cynognathus* are also present in some other cynodonts for which the maxillary canal system has been described, although these have been interpreted as belonging to the EN ramus (Benoit et al. 2020; Pusch et al. 2024). In *Cynognathus*, the two ramifications are clearly distinct from the EN ramus. Since they are directed toward the orbit and arise from the main trunk of the maxillary canal posterior to the EN ramus, we relate them to the topologically similar inferior palpebral (IP) ramus of the mammalian maxillary nerve (sensu Rodella et al. 2012).

Ventral to the IP rami and above the anterior half of the tooth row, branches attributable to the alveolar canals are visible (Fig. 1A, C, D). As in other cynodonts with described maxillary canal systems (Benoit et al. 2020; Kerber et al. 2024a; Pusch et al. 2024), the most anterior of these two canals in both NHMUK PV R 3604 and BP/1/3755 is here interpreted as the RA canal, as it is oriented anteroventrally toward the canine (Fig. 1A, C, D). The RA canal is represented by two branches that diverge separately from the main trunk of the maxillary canal in NHMUK PV R 3604, but are fused proximally in BP/1/3755. The RA canals open externally into two foramina in BP/1/3755 and four in NHMUK PV R 3604. In the reconstructed right maxillary canal system of BP/1/3755, the RA canals are clearly rooted in the main trunk of the maxillary canal (as is common in most cynodonts, Benoit et al. 2020; Kerber et al. 2024a; Pusch et al. 2024; Fonseca et al. 2024), whereas in the left one, the RA canals appear to diverge from the MS or immediately anterior to the MS (Fig. 1C, D; see discussion below). The MA canal is a simple canal that diverges directly from the MS at the level of the fifth (BP/1/3755) or sixth (NHMUK PV R 3604) upper postcanine tooth. It connects it to a foramen on the surface of the maxilla that opens above the fourth postcanine tooth in both specimens (Fig. 1A, C, D). No CA canal could be identified in either BP/1/3755 (because this area is not preserved) or NHMUK PV R 3604 (likely because of the low resolution of the CT scan).

The MS extends from between the level of the third and fourth postcanines anteriorly to just beyond the posterior end of the upper tooth row (Fig. 1A, C, D). It is completely preserved in BP/1/3755 only, where it measures 62 mm in anteroposterior length. Compared with the preorbital length of the snout in this specimen (130 mm), this yields an MS-to-preorbital length ratio of 0.477, which is similar to that measured in *Cricodon* and *Boreogomphodon* (Pusch et al. 2024).

### *Diademodon tetragonus*

The maxillary canal appears overall less branched and proportionally longer in *Diademodon* than in *Cynognathus* (NHMUK PV R 3604), but it is generally organised similarly (Fig. 1B). Anterior to the canine, the IN ramus comprises two short branches, one oriented anterodorsally and the other anteriorly. Ventral to these, the SL ramus has three proportionally longer branches, all oriented anteroventrally toward foramina that open anterior to the canine (Fig. 1B). The EN ramus does not project as far dorsally as in *Cynognathus*, in which it extends above the root of the upper canine (Fig. 1A, B). It is located at the level of the canine and includes only two branches, one oriented anterodorsally and the other posterodorsally. Posteriorly, at the level of the fourth upper postcanine alveolus, a single IP ramus diverges dorsally from main trunk of the maxillary canal (Fig. 1B). The distance separating it from the MS is almost twice as long as that which separates it from the EN ramus. Ventrally, above the level of the sixth upper postcanine alveolus, the RA canal branches off directly from the main trunk of the maxillary canal, although not far anterior to the MS (Fig. 1B). The RA canal divides anteriorly into two branches, one more dorsal than the other, directed toward the upper canine and third upper postcanine alveoli, respectively (Fig. 1B). The MA and CA canals both originate from the MS. The former diverges from the MS at the level of the ninth upper postcanine tooth, and extends anteriorly to a foramen located above the seventh postcanine anteriorly (Fig. 1B). The CA canal is almost vertical and located above the tenth upper postcanine (Fig. 1B). The MS is anteroposteriorly more expansive than that of *Cynognathus*. It extends anteriorly from the level of the eighth upper postcanine alveolus to the base of the jugal arch posteriorly (visible in the CT data of BP/1/3757 on the left side only), extending well beyond the level of the last upper postcanine. The MS measures 73 mm in anteroposterior length against a preorbital snout length of 164 mm, yielding a MS-to-preorbital length ratio of 0.455, very similar to that in *Cynognathus*. 

## Discussion and conclusions

Overviews of the currently available data on the evolution of the maxillary canal system in more crownward Cynognathia are already available (Franco et al. 2021; Medina et al. 2025; Roese-Miron et al. 2025)¸ therefore, this contribution focuses on the implications of the new data about the basal most cynognathians *Cynognathus* and *Diademodon*. These concern character changes of the IP ramus and MS.

**Fig. 2:**
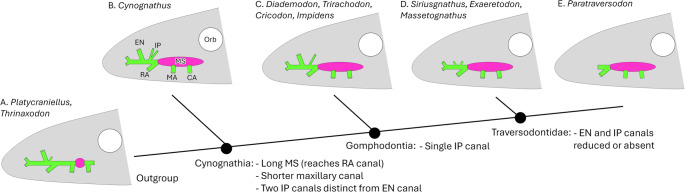
Synthesis of the evolution of the maxillary canal and sinus in Cynognathia. **A**, outgroup; **B**, condition in *Cynognathus*; **C**, condition in basal Gomphodontia; **C**, condition in traversodontids; **E**. Condition in *Paratraversodon* (Crompton et al. 2015; Benoit et al. 2016, 2020; Tolchard et al. 2021; Pusch et al. 2024; Kerber et al. 2024b; Medina et al. 2025; Roese-Miron et al. 2025). Note that the condition in *Siriusgnathus *and* Exaeretodon *reflects the most recent interpretation by Roese-Miron et al. (2025) rather than Franco et al. (2021), and that the condition in *Paratraversodon* is based on the identification of foramina by Kerber et. (2024b). Phylogenetic relationship based on Medina et al. (2025)

In many therapsids with described maxillary canal systems, canals found in a position comparable to the IP ramus are present but difficult to distinguish from the EN ramus because both rami originate in close proximity on the maxillary canal (Benoit et al. 2016, 2018, 2020; Pusch et al. 2024). In contrast, they are sufficiently distinct in *Cynognathus* and *Diademodon* to be confidently identified as such (Fig. 1). A canal that may correspond to the IP ramus is present in *Galesaurus* and *Platycraniellus*, but is arguably absent in *Thrinaxodon* and the Probainognathia, the latter representing the sister-clade to the Cynognathia (Benoit et al. 2016; Kerber et al. 2024a; Pusch et al. 2024; Fonseca et al. 2024). In *Cynognathus*, the basal most cynognathian, the IP ramus is represented by two canals (Fig. 1A, C, D). By contrast, non-travesodontid gomphodonts such as *Diademodon*, *Trirachodon*, *Cricodon*, and *Impidens* retain a single IP canal (Fig. 1B; Benoit et al. 2020; Tolchard et al. 2021; Kerber et al. 2024a, 2024b), whereas in the traversodontids *Siriusgnathus*, *Exaeretodon*, *Boreogomphodon*, and *Paratraversodon*, the IP ramus is absent and the EN ramus is either reduced or absent (Kerber et al. 2024b; Medina et al. 2025; Roese-Miron et al. 2025). This contrasts with the condition present in the traversodontid *Massetognathus*, in which a short, thin but distinct canal is still present in the position of the IP ramus (Crompton et al. 2015; Medina et al. 2025). Altogether, we hypothesise that the IP ramus was either absent or variably present as a single, small canal in the outgroups of Cynognathia. Subsequently, *Cynognathus* evolved two distinct and prominent IP canals (Fig. 2), which were later reduced and eventually lost in traversodontid gomphodonts (Fig. 2).

Benoit et al. (2020) were the first to note that the maxillary canal appears proportionally shorter in *Trirachodon* than in other cynodonts because of the anteroposterior expansion of the MS, with the three alveolar canals originating from the MS rather than having separate points of origin on the maxillary canal. Subsequently, Tolchard et al. (2021), through comparisons with *Cricodon* (erroneously referred to as *Trirachodon*), *Massetognathus* and *Impidens*, suggested that this condition may represent a synapomorphy of the Cynognathia. This hypothesis was later tested through the scoring of corresponding characters into cladistic analyses focusing on cynodont phylogeny (Benoit et al. 2022; Pusch et al. 2024). However, because the conditions in *Cynognathus* and *Diademodon* had not yet been documented, these studies could not determine whether this condition represented a synapomorphy of Gomphodontia or, more broadly, of Cynognathia. According to Pusch et al. (2024; character 19), the length of the MS evolved from less than 29.0% of the snout length to at least 42.0% in cynognathians, which is consistent with the values measured here in *Diademodon* (44.5%) and *Cynognathus* (47.7%). This supports the interpretation that this condition is synapomorphic for Cynognathia. The MS subsequently expanded anteroposteriorly in *Cynognathus* and crownward cynognathians for which maxillary canal systems have been described (see Medina et al., 2025; Roese-Miron et al. 2025), incorporating the posterior portion of the maxillary canal, including some of the alveolar rami (Fig. 2).

It can be reasonably hypothesised that in the plesiomorphic condition in Eucynodontia and Cynognathia, the RA and MA canals likely did not originate from the MS, as in the early non-eucynodont cynodonts *Thrinaxodon* and *Platycraniellus* (Fig. 2). The plesiomorphic position of the CA canal in cynognathians remains ambiguous because: (i) this branch could not be identified in *Cynognathus* here, and (ii) the CA canal branches off from the MS in basal probainognathians and many other non-eucynodont cynodonts (e.g., *Procynosuchus*,* Vetusodon*,* Abdaladon*,* Charassognathus*, and *Cynosaurus*), but not in *Thrinaxodon* and *Platycraniellus* (Benoit et al. 2020; Pusch et al. 2024).

In some cynognathians, the origin of the RA canal may be positioned slightly more anteriorly on the maxillary canal, as is present in *Diademodon*,* Boreogomphodon*, *Siriusgnathus* and *Massetognathus* (Kerber et al. 2024b; Roese-Miron et al. 2025). However, the occurrence of both conditions (branching from the MS or directly from the maxillary canal) within a single specimen of *Cynognathus* (BP/1/3755; Fig. 1C, D) suggests that these minor variations are phylogenetically insignificant (perhaps due, for example, to ontogeny, see Pusch et al. 2024).

## Data Availability

All the data is included in the manuscript.
